# Risk Prediction of Severe Bronchopulmonary Dysplasia (BPD) Using the Respiratory Severity Score (RSS) in Extremely Preterm Infants: A Retrospective Study From Saudi Arabia

**DOI:** 10.7759/cureus.56650

**Published:** 2024-03-21

**Authors:** Eslam M Abuelsaeed, Ahmed M Helal, Abdulrahman A Almehery, Badriah G Alasmari, Harrith Elhag, Monica B Pasubillo, Islam A Farghaly, Mohammed Alomari

**Affiliations:** 1 Neonatal Intensive Care Unit, Armed Forces Hospital Southern Region (AFHSR), Khamis Mushait, SAU; 2 Pediatrics, Armed Forces Hospital Southern Region (AFHSR), Khamis Mushait, SAU

**Keywords:** respiratory support, preterm neonate, chronic lung disease of prematurity, respiratory severity score, bronchopulmonary dysplasia

## Abstract

Background

Bronchopulmonary dysplasia (BPD) is a significant complication in extremely preterm infants. Therefore, early diagnosis of BPD is important for planning treatment strategies. In this study, we aimed to assess the predictive efficacy of the Respiratory Severity Score (RSS) in determining severe BPD or death outcomes in very preterm infants.

Methodology

This retrospective study included preterm infants born with a gestational age of ≤30 weeks. The inclusion criteria comprised individuals who were mechanically ventilated (<1 week) during the first four weeks of life. Any patients who died during the first seven days of life were excluded. RSS values were recorded on days 3, 14, 21, and 28 of life. Multivariate logistic regression was used to identify a correlation between RSS and patient outcomes.

Results

A total of 154 infants were included in the analysis, of whom 82 (53.24%) developed severe BPD and 38 (24.67%) died. RSS was higher in patients who either died or developed severe BPD compared to those who survived. The multivariate logistic regression analysis revealed that RSSs at postnatal day 14 (odds ratio (OR) = 3.970; 95% confidence interval (CI) = 1.114-14.147; p < 0.05), day 21 (OR = 6.201; 95% CI = 1.937-19.851; p < 0.05), and day 28 (OR = 8.925; 95% CI = 3.331-28.383; p < 0.05) was significantly associated with a higher risk of death or severe BPD.

Conclusions

The findings of the present study revealed that RSS can help predict the risk of severe BPD in very preterm infants.

## Introduction

Abnormal lung development is a significant concern in preterm infancy, with bronchopulmonary dysplasia (BPD) being the most common complication [1]. The definition of BPD was first proposed in 1967; however, it has undergone various changes since then. Currently, the National Institute of Child Health and Human Development’s (NICHD) severity-based classification has been widely adopted in clinical practice [2]. Based on their recommendations, BPD is stratified into three different severity stages, namely, mild, moderate, and severe. Previously, another classification was proposed in 1992 based on etiological factors in Japan. However, it could not gain widespread acceptability and is limited to only Japan [3]. The prevalence of BPD varies widely in major cohort studies, ranging from 11% to 50% [4]. This variation is attributed to differences in the criteria used for BPD diagnosis such as gestational age of the infant or weight at birth. The prognosis of preterm infants with BPD is usually poor, with a high risk of developmental disability in surviving patients [5]. So far, several contributors to BPD have been identified, including genetic predisposition, postnatal infections, mechanical ventilation, and inflammation [6].

For preventing BPD, several strategies have been adopted, including gentle ventilation approaches, postnatal steroids, and the application of surfactants. Despite these facts, the incidence of BPD has been on the rise in the last two decades [7]. The management of BPD is quite complex, often requiring prolonged hospitalizations. Currently, the therapeutic landscape of BPD is not standardized, and the long-term outcomes of these approaches are controversial [8]. For instance, the association between sedatives and poor developmental outcomes hints at being prudent while using these medications in BPD patients [9]. Therefore, it is important to identify factors that can predict the severity of BPD. Early diagnosis of BPD can enable timely therapeutic interventions and prevent the use of unnecessary medications such as corticosteroid use. The Respiratory Severity Score (RSS), calculated by multiplying the mean airway pressure (MAP) by the fraction of inspired oxygen (FiO_2_), is a valuable tool for evaluating respiratory failure in later neonatal stages. This is particularly useful in situations where there are no arterial lines in place to measure oxygenation at a steady state, and where obtaining blood gas values through arterial sticks may provide non-steady state results [10].

Previously, various studies have demonstrated the reliability of RSS in determining clinical outcomes in preterm infants. For example, Shah et al. reported that RSS ≥2 at birth was associated with an elevated risk of mortality in preterm infants (birth weight ≥1,250 g) [11]. Furthermore, RSS has also been used as an alternative to oxygen index (OI) for the assessment of patients who require assisted ventilation [12]. A significant correlation has been identified between RSS and OI at arterial oxygen saturation (SaO_2_) levels ranging from 88% to 94% in intubated infants [13]. Although some studies have been published that reported that RSS is a predictor of BPD, there is a paucity of literature published in Saudi Arabia. Therefore, this study was conducted to evaluate the correlation between RSS values at specific intervals and the risk of severe BPD or death in preterm infants.

## Materials and methods

Study design and setting

This retrospective study was conducted in the neonatal intensive care unit at King Faisal Military Hospital between January 2019 and December 2020.

Participants

The inclusion criteria of the study comprised preterm infants with a gestational age of less than or equal to 30 weeks. Furthermore, only patients who were mechanically ventilated (more than one week) during the first four weeks of life were included. Patients having congenital malformations or being intubated for less than seven days were excluded. Patients who died within seven days after birth were also excluded from the study. Furthermore, cases in which systemic steroids were administered and could potentially affect respiratory support were excluded from the analysis.

Data collection

Data regarding demographic details and clinical data about the participants were collected from patients’ charts. Prenatal factors encompassed conditions such as chorioamnionitis, preeclampsia, preterm premature rupture of membranes, and the administration of antenatal steroids. Patients’ demographics included gestational age, weight of the patient, sex, systemic steroid use following delivery, duration of mechanical ventilation, and ventilator settings. RSS values were noted at specific days post-birth (days 3, 14, 21, and 28). To compute the MAP, the following formula was utilized: MAP = PEEP + ((PIP-PEEP) × (ti/ti + te)). PIP stands for peak inspiratory pressure, where te denotes expiratory time and ti represents inspiratory time.

Outcomes

The primary outcome was death or severe BPD. The severity of BPD was evaluated using the criteria established by the NICHD [12]. It was categorized into three levels, namely, mild, moderate, or severe, based on the level of oxygen support needed. Mild BPD indicated breathing without assistance, moderate BPD indicated oxygen support with an FiO_2_ less than 0.30, and severe BPD indicated either an FiO_2_ of at least 0.3 or the need for positive pressure ventilation (PPV) at a postmenstrual age of 36 weeks [12]. Secondary outcomes included assessing the length of time infants required mechanical ventilation or supplemental oxygen, the duration of hospital stay, and other complications such as the use of surfactant for respiratory distress syndrome, the occurrence of grade 3-4 intraventricular hemorrhage (IVH), and retinopathy of prematurity (ROP).

Statistical analysis

The study utilized chi-square and independent t-tests to compare different variables. Additionally, a multiple logistic regression model was employed to analyze the relationships between severe BPD or death and the RSS. Specificity, sensitivity, and positive and negative predictive values were determined using a receiver operating characteristic (ROC) curve. The optimal RSS cut-off was selected using the minimum p-value approach. The statistical analysis was conducted using SAS version 9.4.

Ethics statement

The Institutional Review Board of King Faisal Military Hospital gave the approval for data collection and its use for research purposes before the start of the study (Code: AFHSRMREC/2021/PEDIATRICS/57). Informed consent from the patients was waived by the committee.

## Results

**Figure 1:**
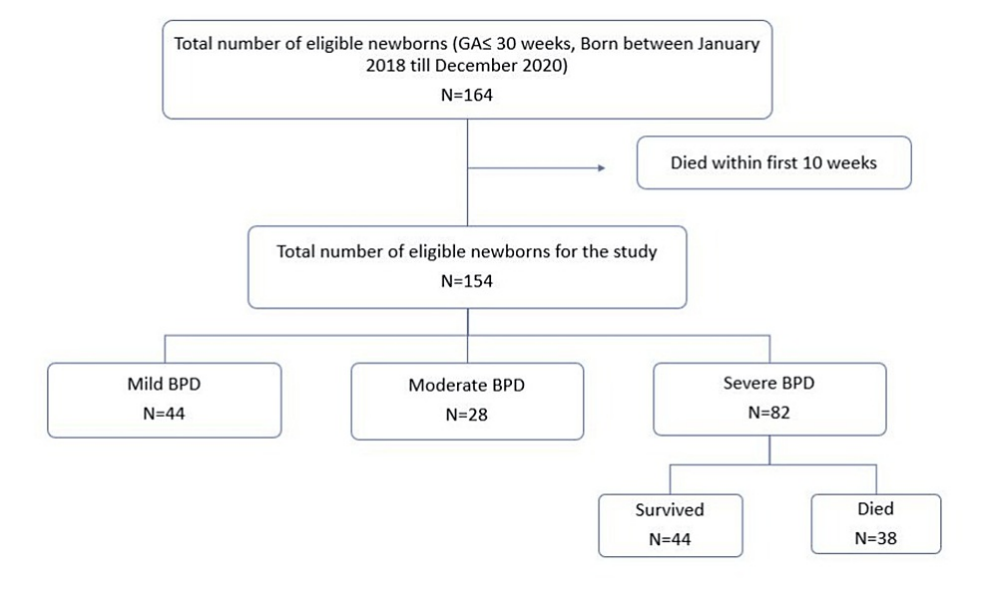
Flowchart of the study population. GA = gestational age; BPD = bronchopulmonary dysplasia

A total of 164 infants met the inclusion criteria. A total of 10 infants died during the first seven days, who were excluded from the study. Out of the 154 included patients, 82 (53.24%) developed severe BPD, and 38 (24.67%) patients died. Figure 1 shows the flowchart of the study.

Table 1 shows the base characteristics of the patients, along with their clinical data. Infants who died or developed severe BPD had significantly lower birth weights, smaller gestational age, increased incidence of PDA, higher incidences of IVH3 or 4, longer durations of assisted ventilation and supplemental oxygen, and longer stay durations than survivors without severe BPD. Other morbidities, such as PROM, chorioamnionitis, preeclampsia, antenatal steroids, administration of surfactant, postnatal steroid ROP stage 3, and sepsis had non-significant differences between the two groups.

**Table 1:** Demographic and clinical characteristics of the study participants. BPD = bronchopulmonary dysplasia; SGA = small for gestational age; NVD = natural vaginal delivery; CS = cesarean section; PPRom = preterm premature rupture of membranes; PDA = patent ductus arteriosus; ROP = retinopathy of prematurity; IVH = intraventricular hemorrhage; MV = mechanical ventilation

Patient details		Group						t-test	
		Without severe BPD			Severe BPD			t	P-value
Gestational age (weeks)		27.948	±	1.739	27.543	±	2.508	1.200	0.232
Birth weight (g)		992.268	±	221.105	851.756	±	191.365	4.351	<0.001*
Apgar score 5		6.756	±	0.937	6.537	±	1.157	1.335	0.184
Chi-square		N		%	N		%	X^2^	P-value
Sex	Female	28		34.15	20		24.39	1.885	0.170
	Male	54		65.85	62		75.61		
SGA		6		7.32	20		24.39	8.959	0.003*
Mode of delivery	NVD	24		29.27	20		24.39	0.497	0.481
	CS	58		70.73	62		75.61		
PPRom		10		12.20	14		17.07	0.781	0.377
Chorioamnionitis		0		0.00	0		0.00	-	-
Preeclampsia		6		7.32	14		17.07	3.644	0.056
Antenatal steroids		50		60.98	58		70.73	1.735	0.188
Surfactant		82		100.00	82		100.00	-	-
PDA		70		85.37	82		100.00	12.947	<0.001*
Postnatal steroid		0		0.00	2		2.44	2.025	0.155
ROP stage 3		8		9.76	16		19.51	3.124	0.077
IVH 3 or 4		4		4.88	18		21.95	10.289	0.001*
t-test								t	P-value
MV duration		18.098	±	7.363	66.415	±	48.860	-8.855	<0.001*
Length of stay		84.512	±	41.499	156.805	±	87.001	-6.791	<0.001*

Table 2 displays a comparison of RSS values on postnatal days 3, 14, 21, and 28 for two groups. Infants who developed severe BPD consistently exhibited significantly higher RSSs compared to those who survived without severe BPD (p < 0.001 for all).

**Table 2:** Comparison of RSS values at postnatal days 3, 14, 21, and 28 between the two groups. BPD = bronchopulmonary dysplasia; RSS = Respiratory Severity Score

	Group						t-test	
	Without severe BPD			Severe BPD			t	P-value
RSS 3 days	2.505	±	0.617	4.602	±	4.376	-4.298	<0.001*
RSS 14 days	2.256	±	0.736	3.463	±	1.874	-5.312	<0.001*
RSS 21 days	2.536	±	2.516	4.100	±	3.201	-3.338	0.001*
RSS 28 days	2.004	±	0.278	4.654	±	4.130	-4.789	<0.001*

After accounting for both prenatal and postnatal factors linked to the development of BPD, the results of the multivariate logistic regression analysis showed that the RSSs on postnatal days 14, 21, and 28 were still significantly correlated with an increased likelihood of death or severe BPD. The odds ratios (ORs) were 3.970 (95% confidence interval (CI) 1.114-14.147) for day 14, 6.201 (95% CI = 1.937-19.851) for day 21, and 8.925 (95% CI = 3.331-28.383) for day 28 (Table 3).

**Table 3:** Multivariate logistic regression analysis of RSSs and association with risk of death or severe BPD. BPD = bronchopulmonary dysplasia; RSS = Respiratory Severity Score; CI = confidence interval

	Odds ratio	95.0% CI for Odds ratio	P-value
RSS 3	1.155	0.427-3.123	0.777
RSS 14	3.970	1.114-14.147	0.033*
RSS 21	6.201	1.937-19.851	0.002*
RSS 28	8.925	3.331-28.383	0.001*

**Figure 2:**
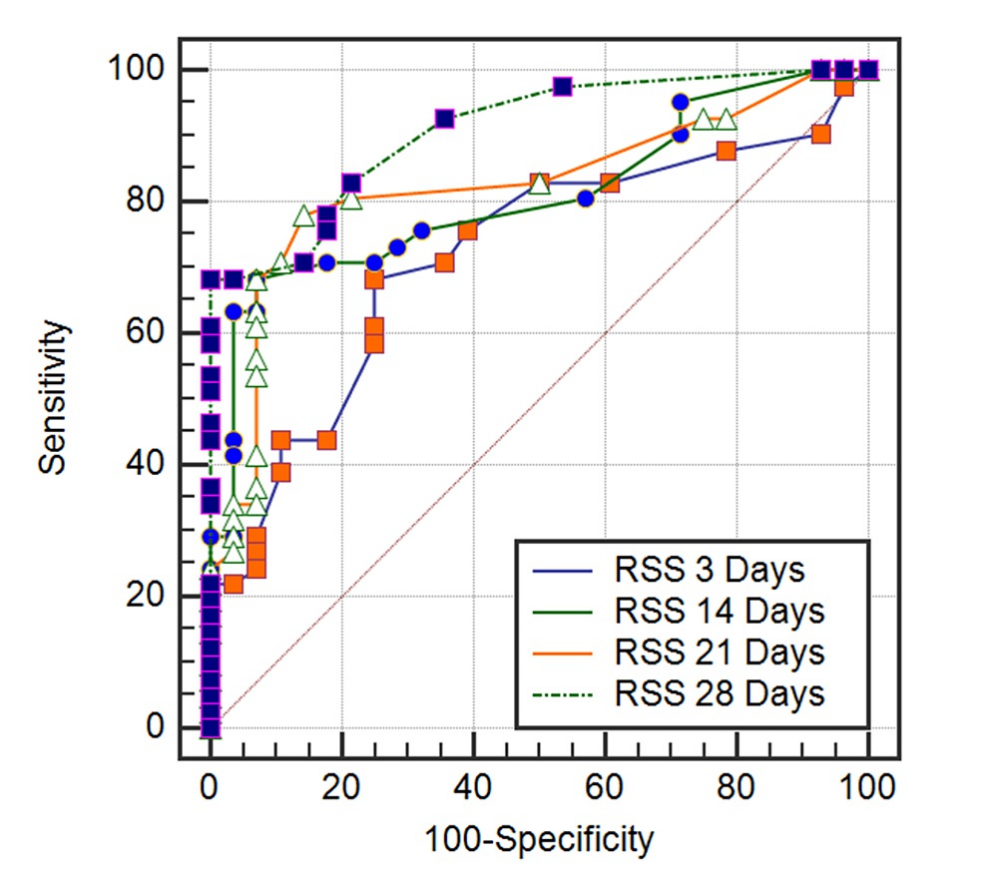
ROC curve between severe BPD and without severe BPD. BPD = bronchopulmonary dysplasia; ROC = receiver operating characteristic; RSS = Respiratory Severity Score

Table 4 and Figure 2 show data for the ROC curve (sensitivity, specificity, positive and negative predictive values of each RSS value in 3,14,21,28, days) between severe BPD and without severe BPD.

**Table 4:** ROC curve between severe BPD and without severe BPD. BPD = bronchopulmonary dysplasia; RSS = Respiratory Severity Score; ROC = receiver operating characteristic; PPV = positive predictive value; NPV = negative predictive value; AUC = area under the curve

	Cutoff	Sensitivity	Specificity	PPV	NPV	AUC	P-value
RSS 3 days	>2.5	68.29	65.85	66.7	67.5	0.687	<0.001*
RSS 14 days	>2.6	68.29	87.18	84.8	72.3	0.781	<0.001*
RSS 21 days	>2.2	78.05	83.33	84.2	76.9	0.805	<0.001*
RSS 28 days	>2.6	68.29	100.00	100.0	68.3	0.909	<0.001*

## Discussion

The findings of this study indicate that RSSs measured at postnatal days 14, 21, and 28 are significantly linked to the risk of death or severe BPD. The RSS values measured on day 14 could be particularly useful for early identification of infants at a heightened risk of developing severe BPD. The RSS measured on day 28 has the highest OR of predicting severe BPD or death. Therefore, RSS can serve as a significant predictor for risk assessment of severe BPD.

In this study, out of 82 patients with severe BPD, 38 (46.34%) died. These findings are much higher compared to some previous studies. Jung et al., in their study, reported that only 14.03% of patients died who developed severe BPD [12]. Furthermore, they reported that 47.8% of eligible patients either developed severe BPD or died. This finding is comparable to our study as 53.24% of eligible patients either died or developed severe BPD in the present study. The severity of BPD was assessed based on new criteria of NICHD proposed in 2019. The new definition of BPD severity categorizes BPD based on the mode of respiratory support administered to very preterm infants at 36 weeks postmenstrual age (PMA) [14]. Some studies have reported that 16% of infants born below the age of 32 weeks PMA ultimately develop severe BPD [15].

Another significant finding of this study was that infants who died or developed severe BPD had significantly lower birth weights and gestational age. These findings are supported by Vakrilova et al. who reported that very preterm infants with lower gestational age and birth weights are at higher risk of BPD. They further revealed that each gestational week resulted in a decrease in BPD by 60% [16]. Similar findings have been reported by other studies as well [17,18], indicating that lower gestational age and birth weights are independent risk factors for BPD.

The management of BPD in extremely preterm infants is quite complex. As not many effective treatment options are available, the main focus of treatment is to reduce the need for ventilation via endotracheal tubes [19]. This approach aims to lower the risk of long-term lung and brain damage in preterm infants. However, some studies have reported that patients with BPD are at an increased risk of having poor neurodevelopmental outcomes, even without definite brain injuries such as intraventricular hemorrhage or hypoxic-ischemic encephalopathy [20]. Lung injury in preterm infants is often managed by avoiding intubation during the first few minutes of life. In such cases, non-invasive respiratory support is recommended [21]. Rutkowska et al., in their study, reported that non-invasive continuous positive airway pressure initiated in the delivery room was protective in BPD [22].

This study also reported that patients with severe BPD require longer durations of assisted ventilation and supplemental oxygen and longer stay durations. Prolonged duration of ventilation remains a significant factor in severe BPD. A multicenter study reported that the median duration of mechanical ventilation in moderate-to-severe BPD was 26 days whereas the mild BPD group had a median duration of only six days [23]. Lapcharoensap et al., in their study, reported that patients with BPD have a high length of hospitalization compared to those without BPD (103 days vs. 66.5 days) [24]. In this study, approximately 70% of mothers were given antenatal corticosteroids. Although we did not assess the effect of this on patient outcomes, several studies have published conflicting evidence. For example, Rutkowska et al. did not find any protective effects of antenatal corticosteroids on severe BPD [22]. However, Travers et al. reported that antenatal corticosteroid use can reduce moderate-to-severe BPD by 43% [25].

A scoring system should ideally be practical, applicable early in hospital admission, and provide reliable estimates of morbidity, mortality, and cost-effectiveness across all newborn patient groups. Previous research on RSS has shown its ease of use, predictive value for morbidity and mortality, and utility in the early stages of life [12,26]. This study has demonstrated that RSS values can be used at 14, 21, and 28 days to predict the severe BPD and mortality risk. Similar to our study, Dursun et al. reported that RSS values are elevated during the first three days of life in infants suffering from severe BPD [27].

Limitations

There are some limitations of the study which should be kept in mind while interpreting the findings of the study. The study included infants requiring intubation for reasons other than lung conditions, such as airway problems or prematurity-related apnea. Second, the analysis was retrospective and drawn from experiences within a single unit. Therefore, it is imperative to conduct further research using a larger prospective cohort study to confirm the utility of RSS in predicting outcomes at a PMA of 40 weeks.

## Conclusions

The present study demonstrates the predictive value of RSS in assessing the risk of severe BPD and death in very preterm infants. The results showed that RSS values can be obtained in the second, third, and fourth weeks of life to accurately determine severe BPD risk. By leveraging RSS assessments during the critical early weeks of life, healthcare providers can identify at-risk infants. Although this study offers valuable insight into the potential of RSS in diagnosing BPD, further prospective studies are warranted to validate these findings and refine the clinical utility of RSS in this vulnerable patient population.
